# Operative strategies and outcomes for patients with severe pulmonary artery hypertension and intestinal obstruction: case series from single institution—sharing lessons to improve surgical outcomes

**DOI:** 10.1093/jscr/rjae711

**Published:** 2024-11-19

**Authors:** Khairunnisa Che Ghazali, Ann Dasimakamalia Mat, Huzairi Yaacob, Muhammad Urfi Omar Hamdan, Ahmad Shanwani Mohamed Sidek

**Affiliations:** Department of Surgery, Hospital Raja Perempuan Zainab II, 15586 Kota Bharu, Kelantan, Malaysia; Department of Surgery, Hospital Raja Perempuan Zainab II, 15586 Kota Bharu, Kelantan, Malaysia; Department of Surgery, Hospital Raja Perempuan Zainab II, 15586 Kota Bharu, Kelantan, Malaysia; Department of Surgery, Hospital Raja Perempuan Zainab II, 15586 Kota Bharu, Kelantan, Malaysia; Department of Surgery, Hospital Raja Perempuan Zainab II, 15586 Kota Bharu, Kelantan, Malaysia

**Keywords:** pulmonary hypertension, Eisenmenger syndrome, intestinal obstruction, emergency surgery

## Abstract

Pulmonary hypertension is a known perioperative risk factor that carries a high morbidity and mortality rate. Severe pulmonary hypertension is related to high morbidity after general anaesthesia. We are reporting three patients with underlying severe pulmonary hypertension, who presented with intestinal obstruction managed with different perioperative approaches. In case 1, a 38-year-old man with Eisenmenger syndrome and severe pulmonary hypertension underwent exploratory laparotomy, right hemicolectomy, and double barrel stoma for obstructed right-sided colonic tumour. He passed away on Day 6 post-operation. In case 2, a 52-year-old man with Eisenmenger syndrome and severe pulmonary hypertension presented with obstructed rectosigmoid tumour and jejunojejunal intussusception and underwent exploratory laparotomy and Hartmann’s procedure. He succumbed after 33 days of fighting with cardiovascular and respiratory complications. In case 3, a 65-year-old woman, with strangulated paraumbilical hernia, underwent mini laparotomy, small bowel resection, primary anastomosis, and paraumbilical hernia repair under monitored sedation and local anaesthesia. She was discharged home after 7 days of hospitalization.

## Introduction

Surgeries carry significant risk for patients with pulmonary hypertension. We may anticipate a worse outcome in patients with significant pulmonary hypertension undergoing non-cardiac surgery under general anaesthesia. Awareness of pre-operative clinical variables may assist clinicians to provide better judgement for patients with severe pulmonary hypertension undergoing non-cardiac surgeries and proactive post-operative measures in these high-risk groups.

## Case summaries

### Case 1

A 38-year-old man had underlying ventricle septal defect with Eisenmenger syndrome since childhood. His echocardiogram showed ejection fraction of 55% and pulmonary artery systolic pressure (PASP) of 121 mmHg. He was on anti-failure frusemide 20 mg OD and cardiprin 100 mg OD.

**Figure 1:**
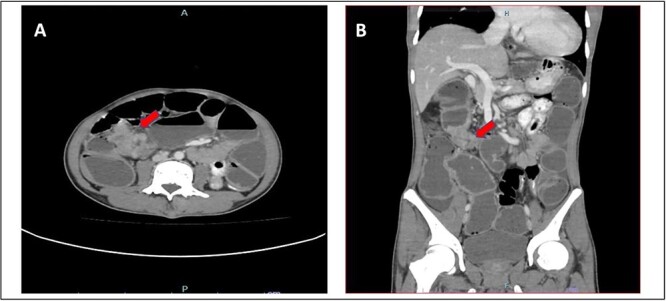
(A) Axial view and (B) coronal view of computed tomography showed short segment thickening of proximal transverse colon (red arrow) with dilatation of bowel.

He presented with a history of abdominal distension for 1 week, loose stool, and vomiting for 3 days. Since admission to the ward, he had no bowel opening and did not pass flatus. On examination, he appeared dehydrated, and his abdomen was soft but distended. Plain abdominal radiograph showed dilated bowel. Computed tomography of the abdomen and pelvis showed short segment thickening of proximal transverse colon with dilatation bowel proximally, enlargement of mesenteric nodes, and mild ascites (Fig. 1). We proceeded with exploratory laparotomy, right hemicolectomy, and double barrel stoma. Intraoperatively, a noted tumour at proximal transverse colon size of about 5 × 4 cm with proximal bowel dilatation and distally bowel collapsed. Multiple mesocolic nodes were also present.

Unfortunately, intraoperatively, he was not stable and needed inotropic support. His post-operative period was stormy. He had acute kidney injury with metabolic acidosis requiring dialysis and haemodynamic instability with maximum inotropic support. He also developed coagulopathy and thrombocytopenia requiring blood products transfusion. His condition deteriorated and on Day 6 post-operation and succumbed to death.

### Case 2

**Figure 2:**
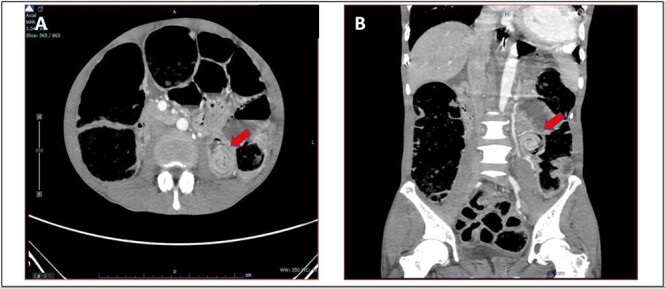
(A) Axial view and (B) coronal view of computed tomography showed presence of doughnut sign at left lumbar region suggestive of jejunojejunal intussusception (red arrow).

**Figure 3:**
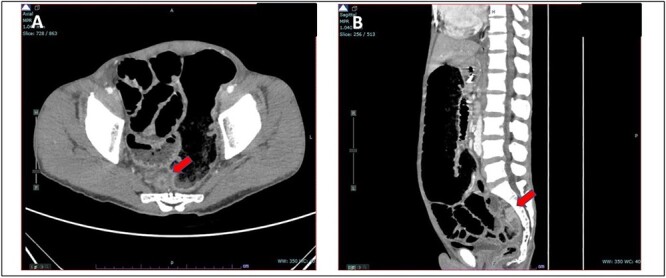
(A) Axial view and (B) sagittal view of computed tomography showed short segment circumferential enhancing bowel wall thickening at rectosigmoid region causing intraluminal obstruction (red arrow).

A 52-year-old Malay man, an active smoker, with underlying pulmonary hypertension secondary to atrial septal defect secundum with Eisenmenger syndrome and chronic lung disease. He had been experiencing symptoms of intestinal obstruction for a week. He had abdominal pain for 1 day, vomiting for 2 days, and unable to pass motion for the past 1 week. Patient was tachypnoeic with a respiratory rate of 35/min, a pulse rate of 116 bpm, and SPO_2_ of 90% under high-flow nasal cannula 50%/50 L. Abdominal radiograph showed dilated large bowel. Computed tomography abdomen and pelvis revealed short segment circumferential enhancing bowel wall thickening at rectosigmoid region causing intraluminal obstruction and presence of target sign at left lumbar region suggestive of jejunojejunal intussusception (Figs 2 and 3).

The patient was then kept nil by mouth on Ryle’s tube free flow and admitted to the intensive care unit for pre-operative optimization with the initiation of broad-spectrum antibiotics, cefoperazone and metronidazole. Urgent echocardiogram performed showing left ventricular ejection fraction of 38%, global hypokinesia, atrial septal defect of size 1.0–2.3 cm with left to right shunt, moderate tricuspid regurgitation with systolic pulmonary artery pressure of 64 mmHg, and moderate mitral regurgitation. The left atrium, right atrium, and right ventricle were all dilated.

Multidisciplinary team discussion was attended by the surgeons, anaesthesiologist, and cardiologist, and the decision was informed to the family members. The anaesthesia and surgery carry a high risk of mortality and morbidity since patient with severe pulmonary hypertension is vulnerable to slight changes in their haemodynamics during surgery. Family member agreed to the operation with high-risk consent.

Hartmann’s procedure was successfully done within an hour. The operative findings were constricting rectosigmoid tumour causing intestinal obstruction, and there were no jejuno-jejunal intussusception identified.

Post-operatively, the stoma was healthy and functioning. We were able to establish enteral feeding for him. He had turbulent post-operative recovery with multiple unsuccessful extubation due to carbon dioxide narcosis secondary to nosocomial sepsis. His tracheal aspirate persistently grew Burkholderia Cepacian of which it was sensitive to ceftazidime and micafungin. He underwent tracheostomy to assist weaning from ventilation. It was further complicated with ventilator-induced diaphragmatic dysfunction and deterioration of cardiac reserve function with repeated echocardiogram showed an ejection fraction of 21% and poor left ventricle function. He succumbed on post-operative Day 33.

### Case 3

A 65-year-old Siamese lady, with underlying chronic rheumatic heart disease and severe pulmonary hypertension, had liver congestion with ascites secondary to right heart failure of which she was on frusemide and sildenafil. Echocardiogram in January 2023 showed ejection fraction of 54%, severe mitral regurgitation and severe tricuspid regurgitation, and PASP of 95 mmHg.

**Figure 4:**
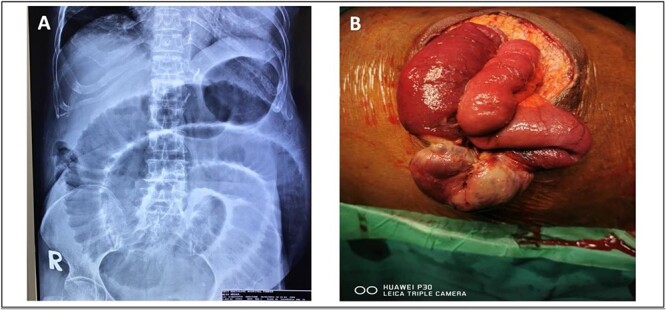
(A) Plain abdominal radiograph showed dilated small bowel (B) intraoperative finding of unhealthy sloughy small bowel segment in the hernia sac and pus was present.

She was presented with an umbilical swelling for the past 1 week which had become painful for the past 2 days, associated with vomiting and unable to pass flatus and bowel motion. On examination, her vitals were normal. Per abdomen, there was 4 × 4 cm swelling at umbilicus with redness and skin changes. Plain abdominal radiograph showed dilated small bowel (Fig. 4A). Her blood parameters showed leukocytosis with white cell counts of 20, haemoglobin of 15, acute kidney injury with urea of 17, and creatinine of 154. Arterial blood gas was normal and no acidosis. The provisional diagnosis was strangulated paraumbilical hernia.

We discussed with patient and her relative regarding the option of anaesthesia either local or general anaesthesia. They agreed and consented for local anaesthesia since it has the least complications and aware of conversion to general anaesthesia if the former method failed to work. We proceeded with the surgery under local anaesthesia and monitored sedation with the help of an anaesthesiologist. We did an infraumbilical transverse incision over the hernia and found small bowel ischemia within the hernia sac with pus (Fig. 4B). The hernia defect was 3 × 3 cm. Ten centimeters of segment small bowel was resected with primary side to side bowel anastomosis using linear stapler 75 mm. The hernia defect is primarily closed with nonabsorbable polypropylene suture. The skin was left open for wound dressing. She recovered slowly from surgery and was able to go home on Day 7 post-operation.

## Discussion

Pulmonary hypertension is one of the most severe complications of congenital heart disease. According to current guidelines, pulmonary hypertension is defined as mean pulmonary artery pressure (mPAP) ≥ 25 mmHg. Eisenmenger syndrome develops in 3.5%–7% of patients with congenital heart disease and pulmonary hypertension [1, 2].

Pulmonary hypertension is a known perioperative risk factor that carries a high morbidity and mortality rate. We may anticipate a worse outcome in patients with significant pulmonary hypertension undergoing non-cardiac surgery under general anaesthesia [3]. These patients are particularly vulnerable to hemodynamic changes brought on by the anaesthesia and the surgery [4]. When non-cardiac surgery is deemed required, the risk to benefit ratio should be considered. Typically, general anaesthesia results in decreased systemic vascular resistance, aggravating right-to-left shunt. In patients with Eisenmenger syndrome, desaturation and hypotension may exacerbate. Therefore, surgeries should be performed in centres with experienced anaesthetists [5]. In a study by Gautam *et al.*, patient’s functional class >II, intermediate to high-risk surgery, and general anaesthesia were independent predictors for short-term morbidity for patients undergoing non-cardiac surgery [3].

In the early published study regarding non-cardiac surgery in Eisenmenger syndrome, Ammash *et al.* retrospectively reviewed 24 patients who attended adult congenital heart disease clinic between 1988 and 1996. His study suggested that Eisenmenger patients with severe right ventricular enlargement and dysfunction had a lengthy operation with anticipated large fluid shifts were at risk for perioperative complications. Contrarily, the majority of small procedures, such tubal ligation and cholecystectomy, can be carried out successfully with extra caution if they are performed within a brief period of anaesthesia and without a considerable fluid change [4].

Most of the case reports or studies described patients with pulmonary hypertension or Eisenmenger syndrome undergoing elective non-cardiac surgery. Hence, patients have time for pre-operative assessment and optimization. Although patients will still have risk of morbidity post-operatively. Yuki *et al.*, in his case report, described a patient with sigmoid colon cancer who also had connective tissue disease along with pulmonary artery hypertension with mPAP of 40 mmHg. This patient was optimized 3 weeks before index surgery with intravenous epoprostenol, and his mPAP reduced to 26 mmHg and proceeded with Hartmann’s procedure. The patient had moderate edema of colonic stoma post-operatively which subsequently gradually improved [6].

In another study, Daisuke *et al.* reported about two cases in his centre. In one study, the patient was diagnosed with obstructing sigmoid colon tumour with underlying severe pulmonary artery hypertension (mPAP of 53 mmHg). This patient had metallic colonic stenting first; epoprostenol was initiated and additional tadalfil to control pulmonary hypertension was administered prior to surgery. Second, a patient with upper rectal tumour with mPAP of 47 mmHg also had a month of preoperative optimization. Both patients underwent Hartmann’s procedure. They had marked edema of colonic stoma post-operatively. The patients were discharged home after 36 days and 120 days post-surgery, respectively. Low tie may be preferable in severe pulmonary hypertension to avoid edema by preserving the venous drainage route of sigmoid colon and approach via open surgery rather than laparoscopically. Pneumoperitoneum and reverse Trendelenburg position can increase systemic vascular resistance index and compression of the lung, subsequently causing respiratory and cardiovascular failure in patient with pulmonary hypertension [7].

In our setting, POSSUM (Physiological and Operative Severity Score for the Enumeration of Mortality and Morbidity) score is commonly used as strategy to risk stratify for high-risk individuals. It was simple to use, helped clinicians identify patient at risk, and assisted patients and their families towards decision-making plan. Thus, during the consultation and family conference, patient’s underlying condition and current surgical emergency that warrant surgical intervention were discussed thoroughly, regarding the prediction of morbidity and mortality risk. In each case, the decision-making process was all set up a through multidisciplinary team, and patients and their families also were well informed (including death complication) and consented for such high-risk procedure.

Since we have bad experience when first dealing with the intestinal obstruction case as illustrated in Case 1, therefore during our encounter with the Case 2 patient who had a similar background of Eisenmenger syndrome and severe pulmonary hypertension, we opted for another strategy to reduce the risk of mortality. This gentleman had dual sites of obstruction from the imaging, namely jejuno-jejunal intussusception and constricting rectosigmoid tumour. Therefore, colonic stenting or diversion colostomy could not be an option during initial presentation as it would not solve the intussusception problem, which was proximal lesion. Even though time was essence, the second patient managed to be optimized in intensive care unit and started with prostaglandin analogue to bring down his mPAP. Surgery was kept short and a low tie of inferior mesentery artery was done to avoid oedema. At least, this patient survived for more than 30 days post-operatively, despite eventually succumbing due to nosocomial sepsis.

As for the third case, knowing that from previous cases patients could not overcome extubation, the risk of patient being under general anaesthesia was higher than the surgery itself. Since this case is amendable and doable under local anaesthesia, we opted this approach. The patient survived and was able to be discharged home.

In a systematic review, perioperative management of patients with pulmonary hypertension undergoing non-cardiac and non-obstetric surgery reported 30-day mortality after elective surgery range from 2% to 18% and it increases to 15%–50% for emergency surgery. The complications and death are usually related to right ventricular failure. Risk factors include procedure-specifics and patient-related factors. Therefore, it is crucial to have an appropriate risk stratification and perioperative plan [8].

Even though Eisenmenger syndrome represents a rare population, it has fragile physiology balance and severe multiorgan disorders. If general anaesthesia is required for any non-cardiac surgery, it should be supervised by experienced anaesthetists who are familiar with fragile pathophysiology, narcotics, and fluid balance as this group of patients is associated with high morbidity and mortality [2].

## Conclusion

Lesson learned from these case series is that we must carefully optimize patients’ prior surgery despite its emergency. Reduction in mPAP can improve patients’ outcome. Multidisciplinary team approach was necessary especially liaising with anaesthetist colleagues. Our experiences all related to emergency surgery; hence, the surgery was necessary to be performed despite its high risk. Each and every life is precious. There are new things to learn and improve in the future so that similar mistakes will not happen.
